# Progress Towards Using Linked Population-Based Data For Geohealth Research: Comparisons Of Aotearoa New Zealand And The United Kingdom

**DOI:** 10.1007/s12061-021-09381-8

**Published:** 2021-04-29

**Authors:** R. A. Oldroyd, M. Hobbs, M. Campbell, V. Jenneson, L. Marek, M. A. Morris, F. Pontin, C. Sturley, M. Tomintz, J. Wiki, M. Birkin, S. Kingham, M. Wilson

**Affiliations:** 1 Leeds Institute for Data Analytics, University of Leeds, Leeds, UK; 2 GeoHealth Laboratory, Geospatial Research Institute, University of Canterbury, Christchurch, Canterbury, New Zealand; 3 Health Sciences, College of Education, Health and Human Development, University of Canterbury, Christchurch, Canterbury, New Zealand; 4 School of Earth and Environment, College of Science, University of Canterbury, Christchurch, Canterbury, New Zealand; 5 School of Geography, University of Leeds, Leeds, UK; 6 Geospatial Research Institute, University of Canterbury, Christchurch, Canterbury, New Zealand; 7 School of Medicine, University of Leeds, Leeds, UK; 8 Alan Turing Institute, London, UK

**Keywords:** Data linkage, Collaboration, International, Geohealth, Health geography

## Abstract

Globally, geospatial concepts are becoming increasingly important in epidemiological and public health research. Individual level linked population-based data afford researchers with opportunities to undertake complex analyses unrivalled by other sources. However, there are significant challenges associated with using such data for impactful geohealth research. Issues range from extracting, linking and anonymising data, to the translation of findings into policy whilst working to often conflicting agendas of government and academia. Innovative organisational partnerships are therefore central to effective data use. To extend and develop existing collaborations between the institutions, in June 2019, authors from the Leeds Institute for Data Analytics and the Alan Turing Institute, London, visited the Geohealth Laboratory based at the University of Canterbury, New Zealand. This paper provides an overview of insight shared during a two-day workshop considering aspects of linked population-based data for impactful geohealth research. Specifically, we discuss both the collaborative partnership between New Zealand’s Ministry of Health (MoH) and the University of Canterbury’s GeoHealth Lab and novel infrastructure, and commercial partnerships enabled through the Leeds Institute for Data Analytics and the Alan Turing Institute in the UK. We consider the New Zealand Integrated Data Infrastructure as a case study approach to population-based linked health data and compare similar approaches taken by the UK towards integrated data infrastructures, including the ESRC Big Data Network centres, the UK Biobank, and longitudinal cohorts. We reflect on and compare the geohealth landscapes in New Zealand and the UK to set out recommendations and considerations for this rapidly evolving discipline.

## Background

An individual’s health status is determined by a myriad of factors including personal physiological and genetic predispositions, cultural, social and economic contexts, and the wider environment within which they live and interact (Dahlgren & Whitehead, 1991). The interconnectedness of risk factors has led to an increased recognition of the need for cross-disciplinary approaches to address public health problems. Health geography is concerned with the spatial relationship between hazardous or healthy features and the incidence of disease or ill-health whilst considering social, cultural and political influences (Moon & Sabel, 2019). A hazardous feature may either encourage an unhealthy behaviour or represent a harmful exposure, and conversely a health-promoting feature may encourage a beneficial behaviour or afford protection (Green et al., 2018). Both are, in turn, related to core geographic constructs such as transportation and urbanisation, which themselves sit within a socio-political landscape. Research in health geography encompasses many themes including non-communicable and infectious disease, health service access and utilisation, and environmental influences on health amongst others (Dummer, 2008).

Health geography has the ability to reveal a range of socio-spatial inequalities, both at the intra- and inter-country level. In high income countries such as New Zealand (NZ) and the United Kingdom (UK), spatial health research is reliant on surveillance data including, but not limited to, the prevalence and incidence of disease and its influencing factors, of variation across the population, and of changes over time. These data are often designed for research or monitoring purposes and come from a range of sources such as cross-sectional surveys, including population censuses and longitudinal cohorts. However, in recent years the expense and limited coverage of these data has prompted a re-think of monitoring systems, including the possible cessation of the UK census after 2021 (UK Statistics Authority, 2018). Researchers now seek alternative data sources that can be repurposed for health geography research. For example, health intelligence systems, such as notifiable disease records and cancer registers, which exist for outbreak monitoring and service provision offer useful incidence and prevalence statistics. Administrative health systems data, such as UK Hospital Episode Statistics, can provide a localised view of health and patient interaction with healthcare services, to understand treatment success and disease trajectories. The linkage of these diverse data sources provides an unrivalled opportunity for researchers to perform complex analyses and examine disease and public health co-morbidities over time and space.

Despite the advantages of linked population-based health data for research, there are significant challenges. Among these challenges are ensuring datasets and systems are secure and fit for purpose, providing streamlined access to linked-health data whilst navigating security and ethical concerns, and ensuring transparency between researchers and data providing organisations, whilst maximising impact (Vogel et al., 2019). Innovative organisational collaborations are therefore central to the effective use of linked population-based health data for research. To extend and develop existing collaborations between the UK and NZ, health geographers from the Leeds Institute for Data Analytics (LIDA), at the University of Leeds, visited the University of Canterbury in June 2019. Together, we (the authors of this paper) convened a two-day workshop that aimed to share existing and planned research using novel or linked data sources for geohealth research.

This paper provides an overview of the lessons learned and insight shared during the workshop, including: the collaborative partnership between the Ministry of Health (MoH) and the University of Canterbury’s GeoHealth Lab (GHL) in NZ which has enabled the successful translation of research to health policy, and the novel infrastructure and commercial partnerships enabled through LIDA. To illustrate this further, we draw upon exemplar projects that have aimed to address public health challenges in the NZ context, which despite similarities with many Western populations, is unique in many ways, including its sparse population density (outside of obvious major urban areas such as Auckland) and bicultural ethnic composition. We consider (a) the NZ Integrated Data Infrastructure (IDI) (Statistics New Zealand, 2013) as a case study approach to population-based linked health data, and (b) similar approaches taken by the UK to move towards integrated health data infrastructure and more effective collaborative partnerships, including partnerships with significant investment in novel emerging data sources (discussed in Sect. 2.3). By reflecting on the health geography data landscapes in NZ and the UK, we recognise the successes and challenges to set out recommendations and considerations for future directions in this rapidly developing area of linked geohealth data. We discuss specific examples such as; the IDI, the GHL-MoH partnership, the ESRC Big Data Network centres, alongside other examples of health data resources including UK Biobank and longitudinal cohorts.

## Overview of Collaborative Data Models

The value of social and spatial data associated with health is recognised around the world (Banerjee, 2016; Warren-Gash, 2017). To make the best of such data and apply insight in a meaningful way relies on interdisciplinarity, co-production and effective collaboration within a suitable environment and infrastructure. Such collaborations, collaborative models or alliances exist at numerous levels, from international initiatives down to the local level, with varying levels of success. Here we will discuss an example of a successful national initiative in NZ that has effectively translated research outcomes into policy impact. We then reflect upon partnerships in a UK context, which include research cohort studies and the use of novel (found/re-purposed) data, and how we can learn from each other in a health geography research and policy setting.

### Collaborative Partnerships in New Zealand

Established in 2005, the GeoHealth Laboratory (GHL) is an effective and innovative collaborative partnership at the University of Canterbury in NZ. The GHL is directly funded by the NZ Ministry of Health (MoH), with the University of Canterbury indirectly funding the lab through staff time (e.g. directorship and collaboration with other staff members) and resources. The reciprocal partnership has a focus on practical health research and is designed to benefit both the health sector and academia. It is at the nexus of ground-breaking and policy-relevant geospatial health research. Historically, projects have included indices of access to health promoting neighbourhood factors, access to undesirable neighbourhood destinations, and access to and utilisation of health services (Bowie et al., 2013). More recent outputs include identifying risk factors for women with obesity of childbearing age (Hobbs et al., 2019a), and accessibility to food retailers and socio‐economic deprivation (Wiki et al., 2019). Other examples include relating the visibility of nature, in the form of green and blue space to psychological distress (Nutsford et al., 2016). These projects are uniquely co-designed by policymakers and researchers to tackle the exigent health issues in NZ in specific policy areas. Effective communication between the GHL and the MoH is therefore critical.

To maximise effective reporting to the MoH, the GHL produces short reports designed to communicate complex analyses to a range of audiences, including policymakers who are not experts in the subject. This approach acknowledges that the length and technicality of many academic publications is a barrier to their use by policymakers, who may lack both time and expertise (Davis & Howden-Chapman, 1996). The ability to simplify research and address the ‘so what?’ is an increasingly valuable skill for academics engaged within applied research settings, however this often conflicts with the publication-focused paradigm of academic career progression. By summarising research outcomes into short reports alongside academic publications, the GHL have developed an effective communication strategy that helps to actualise the real-world impact of research.

The GHL uses data provided by the MoH for health-related and policy-relevant projects (Bowie et al., 2013). Among these data resources is the Integrated Data Infrastructure (IDI), a world-leading innovative research database that is maintained and operated by Statistics New Zealand (Stats NZ) (Social Investment Agency, 2017). The IDI is a longitudinal dataset which holds individual and household level microdata from a range of Government agencies (e.g. housing, health, policing), Stats NZ surveys, and non-governmental organisations (Statistics New Zealand, 2013). The IDI is unique in many ways, as not only does it hold information for 9 million individuals who reside or have resided in NZ, it also hosts data for tens of millions of visitors to NZ (Social Investment Agency, 2017; Statistics New Zealand, 2018a, b). The data are linked using deterministic and probabilistic linkage and completely de-identified before being made available for researchers (Statistics New Zealand, 2013). This is possible due to strict adherence to five ‘safes’; safe people, safe projects, safe settings, safe data, safe outputs (Social Investment Agency, 2017). Researchers are trained and vetted before being granted access to the data; furthermore, the IDI must be used in a safe setting such as the secure data laboratory located at the University of Canterbury. Only projects in the public interest, such as those co-designed by the MoH and the GHL, are approved to use IDI data and all outputs are checked by Stats NZ to ensure they are ‘safe outputs’ before publication (Statistics New Zealand, 2017).

The GHL has reported findings to the MoH on a wide range of topics investigating how neighbourhood and national contexts shape health outcomes and inequality (Bowie et al., 2013). Using linked health-data resources, including the IDI, has allowed researchers at the GHL to answer complex research questions, gaining unique insights for the benefit of broader society. While most conventional data sources used to derive evidence supporting health policies often suffer from a lack of demographic and socioeconomic information, new linked microdata allows better integration of available recorded information in order to generate in-depth insight not (or only hardly) possible before. For example, the GHL utilised the IDI to identify population transience (methodology based on earlier research (Jiang et al., 2018)) and the utilisation of health services. This study identified that up to 5.6% of the NZ population, or 250,000 people, are classified as either ‘vulnerable transient’ or ‘transient’. This research was carried out in collaboration with the MoH and Lakes District Health Board (DHB), one of 20 DHBs in NZ and aimed to determine how home address and frequency of address change (transience) can affect long-term health outcomes and health service utilisation. In the Lakes DHB area, population transience was found to be higher (8.3%) than the NZ national average. A higher proportion of Māori people, fewer people in the 20–39 age group compared to the national average, and a relatively high proportion of people living in the most deprived areas (Sheridan et al., 2011), were also found within these transient groups. These findings enabled Lakes DHB to better understand the characteristics of the affected population, use of primary health services, and their accessibility for vulnerable populations (Ministry of Health | Manatū Haoura, 2019). It is still early to examine the direct impact of utilisation of linked population microdata on health policies. Yet even now, the findings have raised new, more targeted questions from national and regional health sector leaders.

The ongoing longitudinal investigation of immunisation rates in NZ is another example of research focused on health service utilisation undertaken at the GHL. This work identifies socioeconomic and demographic determinants of immunisation using the National Immunisation Register, established in 2005. Researchers accessed general information about vaccinated children including area of residence, gender, ethnicity, socioeconomic status of the residence area, and accessibility of health-related services, as well as detailed information on the immunisation trajectory. The study identified significant differences in immunisation coverage. Specifically, that spatial variation remains even when socioeconomic deprivation, demographic variables, health service accessibility, and urban/rural classifications are controlled for. Higher immunisation rates were associated with less socioeconomically deprived areas and the rates of Ambulatory Sensitive Hospital admissions of children (0–4 years) are lower in areas with higher immunisation rates (Marek et al., 2020). Moreover, recent evidence from NZ suggests there are structural, economic and cultural barriers to immunisation (Walker et al., 2019) and healthcare access in general (Hobbs et al., 2019b). These findings facilitate an improved, area-specific understanding of socioeconomic and demographic determinants of immunisation trajectories throughout a child's lifespan. This empirical evidence has informed progress on the MoH priorities that focuses on child wellbeing and better population health outcomes supported by a strong and equitable public health and disability system (Ministry of Health | Manatū Haoura, 2019).

There are multiple data sources that can be accessed by researchers without any additional steps other than downloading the data. In the New Zealand context, Statistics New Zealand manages multiple online services that allow easy data download (NZ.Stat and Datafinder). The GeoHealth Laboratory also has data it is starting to make available. For instance, on its website, it has a road network layer and other data it has processed. This includes nationwide data on what they defined as environmental “goods” (i.e. greenspaces) and “bads” (i.e. alcohol outlets or gambling venues) (Marek et al., Under Review). The location of such environmental “goods” and “bads” have recently been associated with adverse outcomes for mental health and psychological distress in a nationally representative population of New Zealand adults after controlling for key covariates (Hobbs et al., In Press). For health-specific datasets, the Ministry of Health publishes data and reports on the website, however the spatial (and time) domain of the data is not always optimal. In some of the GeoHealth Laboratory projects the data is not ordinarily available but access was facilitated through the collaborative GHL-MoH partnership. The IDI database is different in that it is available to approved New Zealand researchers. The IDI is then accessible to any researcher upon submitting the project that serves for the public good. To become an approved researcher, one needs to undergo training and check by Stats NZ due to accessing and handling potentially confidential data.

In all the projects carried out by the GHL through the partnership the research questions are co-designed with the Ministry of Health and are therefore are of real value to the health sector. It also means that the path between research evidence and policy change is short and direct. For example, for every project a short plain English summary is produced and has to be signed off at a high level within the Ministry of Health, and shown to the Minister of Health. It would be ideal to show how the research has directly impacted policy, but the link is rarely that simple. One example is investigating the link between the location of alcohol outlets and adverse health outcomes. The GHL has done research on this in 2012 (Day et al., 2012) and 2020 (Hobbs et al., 2020); the latter a response for an update to the earlier research. In addition requests for the research come from the public and other parts of government (e.g. a recent request from the Police) and increasingly alcohol outlets are failing to get permission to locate in residential neighbourhoods (e.g.^1^). In another example, the “Transience” study served as evidence in the development of new policies on housing and displaced population. It also supported actions shaping transformations in the healthcare provision in Lakes DHB.

### Collaborative Partnerships in the United Kingdom

The collaborative partnership between the MoH and GHL is one of a handful of initiatives worldwide which has successfully used linked population-based health data (Vogel et al., 2019; Warren-Gash, 2017), to mobilise change. In the UK, there have been attempts to move towards a solution for linked population health and administrative data, akin to the IDI in NZ. To date, no equivalent resource exists. One such initiative was the ‘Big Data Network’ funded by the Economic and Social Research Council (ESRC) in 2013, phase one of which included the Administrative Data Research Network (ADRN) (Economic Social Research Council, 2019). The main aim of this network was to link de-identified data collected routinely by government departments, for example, health records with education, employment and/or crime data, at an individual level. The ADRN had four nodes, representing the four countries within the UK: England, Wales, Scotland and Northern Ireland. Operating for the devolved nations presented different scales of data linkage and different organisational challenges. With England 10 times larger than the UK’s next largest country, Scotland, with 53 million people compared to 5.5 million respectively (Office for National Statistics, 2020), population size between the countries varies widely. NZ, with a population of 5.0 million (Statistics New Zealand, 2020) is akin to that of Scotland. Computational power exists to handle significant data volumes, suggesting this should not be a problem. However, as individual data sources are governed by a larger number of administrative units in bigger countries, data linkage becomes increasingly complex. Moreover, the time and resources required to extract, clean and anonymise the data, without added benefit or funding support, meant there has been a lack of incentive for data owners to share their data (UK Statistics Authority, 2016). As a result, the ADRN has been slow to acquire datasets, in particular, from government departments such as the Departments for Work and Pensions, which did not have the resource for these processes (UK Statistics Authority, 2017). To make matters worse, ADRN worked on a ‘create and destroy’ policy whereby data could not be reused in numerous projects, which was neither a sustainable nor a cost-effective way of sharing data for research.

In light of these challenges, in the second round of funding the ADRN has been re-invented as the Administrative Data Research (ADR) UK. ADR UK is a partnership between the three established ADRN nodes in Scotland, Wales and Northern Ireland, alongside the Office for National Statistics (ONS), which represents England. Taking learnings from the original ADRN, the current ADR UK has recognised the importance of investing in the data preparation process and realised that this should not be the onus of the data owner. The ‘create and destroy’ policy has been eliminated in favour of reusable themed datasets to which trained researchers may apply for access to de-identified data via a secure data centre. ADR UK also models their administrative data on the ‘five safes’ (Social Investment Agency, 2017) and emphasises the importance of partnerships between government departments and academia. However, unlike the NZ strategic partnership in which the research agenda is devised in collaboration between policy makers and academics, under the ADR UK model, research is led by academic interest.

In addition to administrative data resource linkage, there have been successes with incorporating routinely collected data into longitudinal research cohorts at the individual level. For example, the ONS Longitudinal Study (LS) contains linked individual-level census and administrative data across five successive censuses, for a 1% sample of the population of England and Wales (Shelton et al., 2019). Similar to the LS, the Scottish Longitudinal Study and Northern Ireland Longitudinal Study capture the populations of the rest of the UK (Boyle et al., 2009). Over the past 40 years, the LS has collected data for over 1.1 million individuals as new members enter the study through birth and immigration. Information on life events including births, deaths and health outcomes are linked to census records, for example cancer registrations via the National Health Service Central Register. The large sample size in the LS, afforded by utilising census records, enables analysis of small areas or subsets of the population, such as particular ethnic groups or occupational groups, which is not possible using other longitudinal datasets due to insufficient numbers. At a national level, the LS has provided evidence to support major reports for the government on health and mortality (Marmot, 2010) and in academic research on health inequalities over space and time (Blackburn et al., 2013; Johnson, 2011; Murray et al., 2019). The main limitation of the LS for health research is the lack of behavioural and lifestyle data. Regional cohort studies are better able to capture a greater breadth of such health indicators.

One advantage of regional birth cohort studies, such as the Avon Longitudinal Study of Parents and Children (ALSPAC) and Born in Bradford, is their ability to collect primary data on study participants via questionnaires, clinical assessments and biological samples alongside patient consent to link routine data moving forward. This is enabled by relatively small sample sizes. Both studies follow the lives of approximately 14,000 children born between 1991–1992 and 2007–2010 respectively, as well as the lives of their parents and, in the case of ALSPAC, their offspring. In addition, information about health, wellbeing and educational outcomes is collected by data linkage to routinely collected health data from hospitals, GP practices and local government systems recording educational progress. A sense of place is important in such studies and research investigating the relationship between the environment and health is facilitated by geospatial data linkages (Boyd et al., 2019). Study data can be linked with data on the physical and social environment using geocoded records of participants’ residential location across the life course. Local cohorts have the flexibility to capture data which tackles local issues, for example the long term impact of air pollution during pregnancy (Schembari et al., 2015) and the association between exposure to green space and mental health in children (McEachan et al., 2018). This model has proven successful in forging links between communities, health services and local government, maximising local impact.

The UK Biobank has utilised the success of a longitudinal model that collects behavioural, lifestyle and biological data but at a national level, recruiting a sample of 500,000 participants aged 40–69 years. With the aim of improving the prevention, diagnosis and treatment of chronic illnesses, primary participant information is linked to a range of electronic health records. Despite the richness of individual-level data, there are limitations to the use of UK Biobank for spatial analysis. Potential geographical bias and recruitment bias exist due to the location of test centres locations, which may affect generalisation to the wider population (Batty et al., 2019; Fry et al., 2017). Whilst these valuable local, national and regional models represent isolated examples of good practice in the UK, their coverage is not comparable to the IDI in NZ and the process of replicating this data infrastructure at a UK wide scale would be extremely costly.

## Novel and ‘found’ Data in Health Geography Research

Costs associated with recruitment, data processing, linkage and administration amongst other things are barriers to the creation of an individual level data set that is inclusive of the population. Increasingly, organisations and academic institutions are turning to ‘found’ (Timmins et al., 2018) or novel data. Such data are generated on a daily basis from a variety of sources including apps, social media, and wearable devices (Dinh-Le et al., 2019). Moreover, there is a growing recognition that the complexity of health problems requires the utilisation of non-traditional data, which can provide additional social and cultural context. We now discuss some examples of novel and ‘found’ data in the context of obesity, colorectal cancer, and the use of mobile phone data.

Firstly, a recent exercise mapping data against the Foresight Obesity Systems map, identifying more than 100 contributing factors (Butland et al., 2007), concluded that novel ‘found’ data sources, such as retail transactions, physical activity trackers, and surveillance cameras, are required to fill the gaps left by traditional research data (Morris et al., 2018). This work was undertaken by the Leeds Institute for Data Analytics (LIDA), an academic institution, based at the University of Leeds, designed to foster interdisciplinary, cross sector collaborations through both its physical infrastructure, collaborative working environment and data partnerships. The centre has a number of ‘safe rooms’ (UK Data Service, 2020), enabling access to potentially disclosive data, and a secure computer infrastructure which is NHS Toolkit compliant and accredited with information security standard ISO27001 (International Organization for Standardization, 2013), making it one of few with such accolades in the UK. LIDA is home to numerous research council and charity funding awards that promote data analytics, including health geography components. LIDA also hosts the Consumer Data Research Centre. Established as a complementary initiative to the ADRN in the ESRC Big Data network as one of a number of UK centres that go beyond routinely collected administrative data. The Consumer Data Research Centre engages commercial data partners for linkage of novel data sources, such as: retailer loyalty cards, physical activity apps and market research surveys (Sun & Mobasheri, 2017). Ongoing projects include using supermarket loyalty card data to investigate food purchasing behaviours (Clark et al., 2020; Jenneson et al., 2020), social media data as a source of public health surveillance (Oldroyd et al., 2018), and influence of cycle infrastructure on myocardial health outcomes (Munyombwe et al., 2020).

Hosted by LIDA the COloRECTal Repository (CORECT-R) is designed to improve the outcomes of colorectal cancer treatment by linking ‘found’ data from multiple de-identified routine datasets from across the cancer pathway of diagnosis, treatment and outcome (Bowel Cancer Intelligence UK, 2019). Generated by Bowel Cancer Intelligence (BCI) UK, these include existing data from national cancer registries, hospitals, screening programmes, clinical trials and biobanks and will also include novel datasets such as social care, consumer data, social media, housing, and transport in the near future (Bowel Cancer Intelligence UK, 2019). The data is linked at the patient level and aims to promote early diagnosis, optimise treatments, increase efficiency of NHS services and improve outcomes for cancer patients. While data covering different aspects of cancer and its care currently exist, access for researchers to link and utilise these is limited. The CORECT-R repository affords researchers with new opportunities to explore co-morbidities and lifestyle associated risk factors, such as red meat consumption. These examples highlight the continued utility of the traditional cohort study and demonstrate appetite for enhancing these with a wider variety of data including consumer and administrative data sources.

The utility of novel data resources to enhance current data is beginning to be recognised in NZ. The Sensing City project, which connected respiratory patient’s data to air pollution is one such example (Marek et al., 2016). Moreover, aggregated mobile phone movement data, for example from smartphone applications as well as cell tower triangulation, has been particularly effective during the COVID-19 pandemic in understanding the role of transmission and mobility as well as adherence to social distancing guidelines and ‘lockdowns’ in many places (Science Media Centre, 2020). It is perhaps however the success of the collaborative model in NZ (MoH-GHL) and prize linked data source (IDI) that has prevented the need to acutely seek alternative novel data sources. National mobile phone data, aggregated to census areas in NZ, furnishes researchers with a new data source, uncovering links between environments (whether social or physical) and health outcomes or health behaviours, allowing a deeper and richer understanding of the connections between health and place.

## Beyond Data: People Matter

Not only are data and infrastructure essential for successful health geography collaborations, so are people and their expertise. Local settings such as LIDA facilitate this organic formation of cross-disciplinary ideas. They are however required on a larger scale to generate greater impact. For example, the Alan Turing Institute (ATI), a National Institute for Data Science established in 2015, boasts 13 University partners, alongside principal sponsors, strategic partners and non-academic collaborators (The Alan Turing Institute, 2020a). Using a partnership approach, strategic research programmes at the ATI are broad ranging and foster collaborations between experts across various institutions. These thematic workstreams, such as Urban Analytics and Health and Medical Sciences, combine elements of health geography research (The Alan Turing Institute, 2020a). A recent example of successful collaboration includes the Rapid Assistance in Modelling the Pandemic project which seeks to establish new models, rich in new forms of health and social data and the behavioural analytics which are required to inform the government of possible exit strategies from Coronavirus lockdown (The Alan Turing Institute, 2020b). Research programmes at the ATI capitalise on novel and found data sources, which to date NZ’s IDI lacks. Additionally, the collaboration between experts and development of a physical and virtual environment, as exemplified by the ATI, is yet to be fully realised in NZ. In recognition that translating research findings to policy action goes beyond data and infrastructure, the public policy programme at the ATI connects academics and industry stakeholders with policy makers with the aim of solving long-running ‘wicked’ problems, such as obesity.

Traditionally, academic-led projects in the UK run over several years, in contrast with the short-term GHL projects, to align with funding periods. They often focus on novel method development and therefore take longer to translate to policy outcomes and their impact may be less tangible. The UK’s longer-term funding model, alongside the publication-centric way in which academic performance is measured by the Research Excellence Framework (REF) (Terämä et al., 2016), may distract from a focus on the potential short-term societal impacts of research. Instead, many researchers regard the pathway to impact as an afterthought and in conflict with traditional academic methods of dissemination. That said, recent changes to the REF model (Terämä et al., 2016) seek to change this culture and bring impact to the forefront of research design, practice and dissemination. The co-designed short-term GHL-MoH projects meet the government’s need for concise and timely research outputs and the practice of writing short policy-facing reports enables tangible policy change. One recent example has been some urgent COVID-19 work the GHL has undertaken for the MoH using nationwide mobile phone movement data to quantify the effect of an enforced lockdown on population mobility by neighbourhood deprivation (Campbell, 2021) and the second, spatially identified vulnerable populations who may be more at risk from the pandemic (Wiki et al., 2021, 2019). Future areas of development in the area of geohealth could include more research linking data across government agencies (as the IDI allows) and the increased use of personal mobility data, such as that available through mobile phone data (such as the COVID-19 work done in NZ). The collaboration with the Ministry of Health allowed for rapid sharing of data and analyses during the first wave of COVID-19 epidemic in New Zealand. However, there were more academic centres and institutions directly involved in the research in this particular case. Alongside academic publications, this dissemination model constitutes an exemplar framework that could be more widely adopted within academia to contribute towards the impact agenda.

## Concluding Thoughts

Strategic partnerships such as the GHL in NZ and LIDA in the UK provide opportunities, challenges, and considerations for both the collaboration and the use of linked data for health geography research. Building strategic partnerships, and effectively utilising population-based data within these, has the ability to strengthen both academic institutes and government bodies. As such, organisations are fundamentally different; striking a balance between the priorities of both academic institutions and government does not come without challenges. Where academic research generally focuses on the theoretical foundations that underpin knowledge, the government perspective largely centres on the allocation of resources to tackle real-world problems. As such, a particular and often legitimate criticism of academia is its reticence or inability to engage with those beyond the ‘ivory tower’. Without partnerships between academia and policymakers or industry, the impact and translation of research into action can be absent. The GHL and MoH strategic partnership demonstrates that close and regular engagement between partners promotes trust and understanding of organisational culture and process, resulting in a collaborative approach not only in name, but also in nature. The input and expertise from both sides provides new perspectives allowing researchers and organisations to break out of their disciplinary silos to better explore the link between health and place, facilitating meaningful changes in health policy.

## Footnotes

1 https://www.stuff.co.nz/the-press/news/124006448/plan-for-new-christchurch-liquor-store-withdrawn-after-barrage-of-objections
